# A new record of the genus *Mesocrina* Foerster and a newly-recorded species of genus *Alloea* Haliday (Hymenoptera, Braconidae, Alysiinae) from Korea

**DOI:** 10.3897/BDJ.13.e159712

**Published:** 2025-08-26

**Authors:** Juhyeong Sohn, Cornelis van Achterberg, Sangjin Kim, Yerim Lee, Hyojoong Kim

**Affiliations:** 1 Animal Systematics Lab., Department of Biological Science, Kunsan National University, Gunsan, Republic of Korea Animal Systematics Lab., Department of Biological Science, Kunsan National University Gunsan Republic of Korea; 2 Institute of Insect Sciences, College of Agriculture and Biotechnology, Zhejiang University, Hangzhou 310058, Hangzhou, China Institute of Insect Sciences, College of Agriculture and Biotechnology, Zhejiang University, Hangzhou 310058 Hangzhou China

**Keywords:** Alysiini, Hymenoptera, new records, parasitoid wasp, South Korea, taxonomy

## Abstract

**Background:**

The subfamily Alysiinae is a relatively large group within the family Braconidae, comprising more than 2,440 valid species worldwide. It is divided into two tribes, Alysiini and Dacnusini and is characteried by koinobiont endoparasitoidism of dipteran larvae. In South Korea, 286 species from 21 genera have been recorded to date. Despite this diversity, genus *Mesocrina* have not previously been reported from the Korean Peninsula. *Mesocrina* includes species are parasitoids of cyclorrhaphan Diptera inhabiting fungi, while *Alloea* species are known as parasitoids of *Lonchoptera* species. For the genus *Alloea*, only one species has been recorded in Korea. These genera can be distinguished by specific morphological traits, such as wing venation and shape of mandible.

**New information:**

*Mesocrina indagatrix* Foerster, 1863 is recorded for the first time from Korea, and the genus is recorded new for Korea. In addition, *Alloea veles* Belokobylskij,1997 is recorded for the first time in Korea, which represents the first record of *Alloea veles* outside from Russia and the second record of *Alloea* from Korea. The barcode region of mitochondrial *cytochrome c oxidase I* (*COI*) was also analyzed for the species. Also, we provide an identification key for the *Alloea* species recorded from Korea.

## Introduction

The subfamily Alysiinae is a relatively large taxon within the family Braconidae, consisting of more than 2,440 valid species worldwide (Yu et al. 2016). The subfamily Alysiinae is divided into two tribes: Alysiini and Dacnusini, which include 76 and 31 genera, respectively. In Korea, 286 species from 21 genera have been recorded; 148 species belong to Alysiini and 138 to Dacnusini (NIBR 2022). These two tribes can be distinguished by the presence (Alysiini) or absence (Dacnusini) of vein r-m on the fore-wing (Shaw and Huddleston 1991), but it is not clear if all wingless or brachypterous Alysiinae belong to the Alysiini and some genera of Alysiini may belong to the Dacnusini (Godfray and van Achterberg 2024b). Alysiinae belongs to the Cyclostome clade and exclusively comprises koinobiont endoparasitoids of dipteran larvae (Yu et al. 2016). Members of Alysiinae have exodont mandibles with 2–7 outwardly curved or straight teeth, which they apply to emerge from the host puparium (Docavo et al. 2002). Several species within Alysiinae have been utilized for the biological control of dipteran pests in *Liriomyza trifolii* (Diptera, Agromyzidae) and *Drosophila suzukii* (Diptera, Drosophilidae) (Ozawa et al. 2001, Chabert et al. 2012).

The genus *Mesocrina* Foerster, 1863 is a small genus of Alysiinae, which includes eight species (Godfray and van Achterberg 2024a): six from the Palaearctic and two from the Nearctic. *Mesocrina* species are known as parasitoids in cyclorrhaphan Diptera in fungi. This genus can be diagnosed from other Alysiinae genera by the following combination of characters: third antennal segment longer than fourth; in fore-wing, vein r emerging submedially from oval part of pterostigma; pterostigma largely wide oval or triangular; vein 3-CU1 slender and longer than vein CU1b; in hind-wing, vein m-cu present.

The genus *Alloea* Haliday,1833 is a small genus of Alysiinae, which includes 14 species (Yu et al. 2016): six from the Oriental, four from the Eastern Palaearctic, three from Western Palaearctic and one from Nearctic. In Korea, since *Alloea kostroma* Belokobylskij, 1998 was recorded by Papp (2007), no additional species in this genus have been described. *Alloea* species are known as parasitoids in *Lonchoptera* species. This genus can be diagnosed from other Alysiinae genera by the following combination of characters: third tooth of mandible small, acute and shorter than elongate second tooth; in fore-wing, vein 1-SR of fore-wing distinct and in lateral view metanotum with truncate protrusion medio-dorsally; malar suture often rather long and deep.

In this study, we used the *COI*, barcode region of the *Mesocrina indagatrix* and *Alloea veles* to identify the species. Comparative diagnoses are provided to distinguish these newly-recorded species from morphologically similar species. In addition, descriptions, diagnoses, identification keys and photographs of the diagnostic characters are also provided.

## Materials and methods

**Figure 1.**
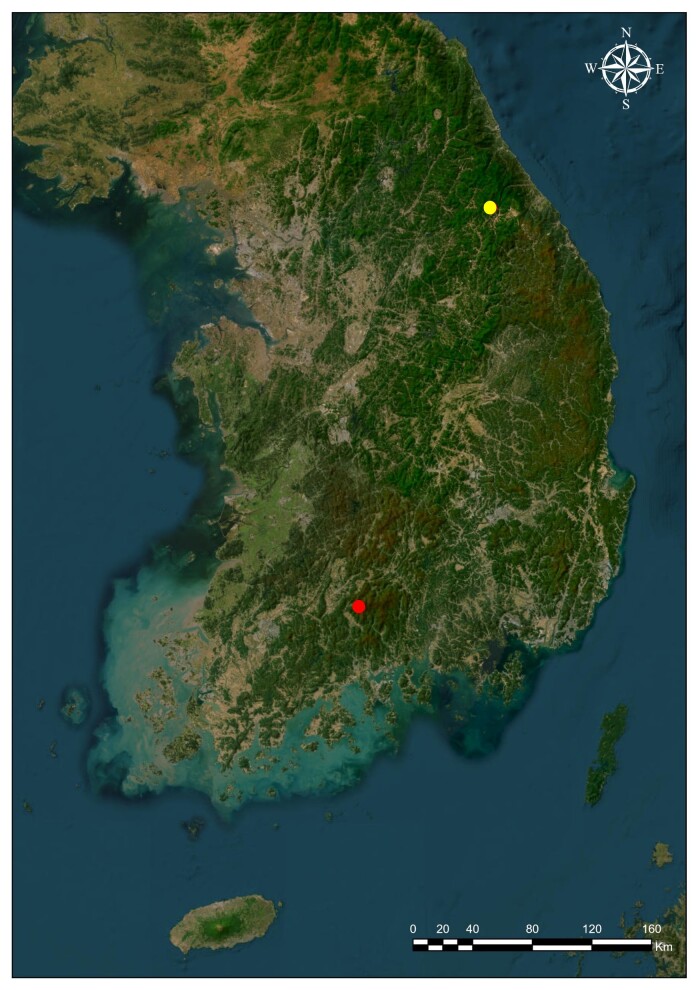
Origin of the newly-recorded species from South Korea. Mt. Odaesan (Gangwon-do) yellow spot; Mt. Segeolsan (Jeollanam-do) red spot.

Samples of the new species were collected using Malaise traps and sweeping in South Korea at Mt. Odaesan (Gangwon-do) and Mt. Segeolsan (Jeollanam-do) (Fig. 1). The sorting and preparation were performed at the Animal Systematics Laboratory (**ASL**), Department of Biological Science, Kunsan National University (**KSNU**).

Whitfield et al. (1997) and Zhu et al. (2017) were used for morphological identification of generic and subgeneric levels. Morphological characters were observed with a Leica M205 C stereomicroscope. The Taxapad database (Yu et al. 2016) was used for references up to 2015. The terminology follows van Achterberg (1993). Specimens of newly-recorded species have been deposited in the **NIBR** (National Institute of Biological Resources, Incheon) collection. A Leica DMC2900 digital camera and a Leica M205 C stereomicroscope (Leica Geosystems AG, Mannheim, Germany) were used for photography; all pictures were taken for each final photo using multi-focusing technology. LAS V4.11 (Leica Geosystems AG, St. Gallen, Switzerland) and Helicon Focus 7 (Helicon Soft, Kharkiv, Ukraine) software were used to stack the photographs. Final illustrations were created using Adobe Photoshop CS6.

For DNA barcode analysis, whole genomic DNA was extracted from the specimens using a Labopass Tissue kit (Cosmo Genetech, Daejeon, Korea) following the manufacturer's protocol. In order to conserve morphologically complete voucher specimens, the ‘non-destructive method’ by Favret (2005) and the ‘freezing method’ by Yaakop et al. (2009) were used, with slight modification to avoid the first crushing of the sample. In the original protocol, the sample was crushed or damaged and then soaked in 180 μl of buffer ATL + 20 μl of proteinase and incubated at 55°C for 3 h. In the slightly modified DNA extraction methods, samples were incubated in 180 μl of buffer ATL + 20 μl of proteinase K without first crushing the sample, followed by a 10-min incubation at 55°C and then kept in a freezer at −22°C overnight. Subsequently, a general protocol was followed for the remaining steps. The primer sets of *LCO-1490* (5'-GGTCAACAAATCATAAAGATATTGG-3') and HCO-2198 (5'-TAAACTTCAGGGTGACCAAAAAATCA-3') was used to amplify approximately 658 bp as the partial front region of the *COI*. The polymerase chain reaction (PCR) products were amplified by using AccuPowerH PCR PreMiⅹ (BIONEER, Corp., Daejeon) in 20 μl reaction mixtures containing 0.4 μM of each primer, 20 μM of the dNTPs, 20 μM of the MgCl_2_ and 0.05 μg of the genomic DNA template. PCR amplification was performed using a GS1 thermo-cycler (Gene Technologies, Ltd., U.K) according to the following procedure: initial denaturation at 95°C for 5 min, followed by 34 cycles at 94°C for 35 sec; an annealing temperature of 48°C for 25 sec; an extension at 72°C for 45 sec and a final extension at 72°C for 5 min. PCR products were visualised using electrophoresis and a 1.5% agarose gel. A single band was observed and sequenced using an automated sequencer (ABI Prism 3730 XL DNA Analyzer, California, USA) at Macrogen Inc. (Seoul, South Korea). Sequence alignment was performed in MEGA version 7 (Kumar et al. 2016) with the ClustalW method. To estimate the pairwise genetic distances, the P-distance model was conducted using MEGA version 7.

## Taxon treatments

### Mesocrina

Foerster, 1863

A4B0CC09-551E-5A1A-B09F-FE01C1D5D4C5

Mesocrina indagatrix Foerster, 1863

#### Diagnosis

Third antennal segment longer than fourth; in fore-wing, vein r emerging submedially from oval part of pterostigma; pterostigma largely wide oval or triangular; vein 3-CU1 slender and longer than vein CU1b; in hind-wing, vein m-cu present.

#### Distribution

Holarctic.

#### Biology (Host)

*Nanna armillata* Zetterstedt, 1846, *Nanna flavipes* (Fallén, 1819) (Königsmann 1959).

### Mesocrina indagatrix

Foerster, 1863

23FE7039-6903-5A58-AF66-868D2350BE77

PV546355

#### Materials

- **Type status:** Other material. **Occurrence:** sex: female; lifeStage: adult; preparations: dry-specimen; associatedSequences: PV546355; occurrenceID: 2ECB2288-B428-5437-8347-B5BA7E074CFC; **Taxon:** scientificName: *Mesocrina indagatrix* Foerster,1863; kingdom: Animalia; phylum: Arthropoda; class: Insecta; order: Hymenoptera; family: Braconidae; genus: Mesocrina; specificEpithet: *indagatrix*; scientificNameAuthorship: Foerster, 1863; **Location:** higherGeography: East Asia; country: South Korea; countryCode: KR; stateProvince: Gangwondo; municipality: Pyeongchanggun; locality: Mt. Odaesan; decimalLatitude: 37.79165833333333; decimalLongitude: 128.5654; **Identification:** identifiedBy: Ju-Hyeiong Sohn; **Event:** year: 2020; month: 9; day: 18; **Record Level:** institutionCode: NIBR

#### Description

**Figure 2.**
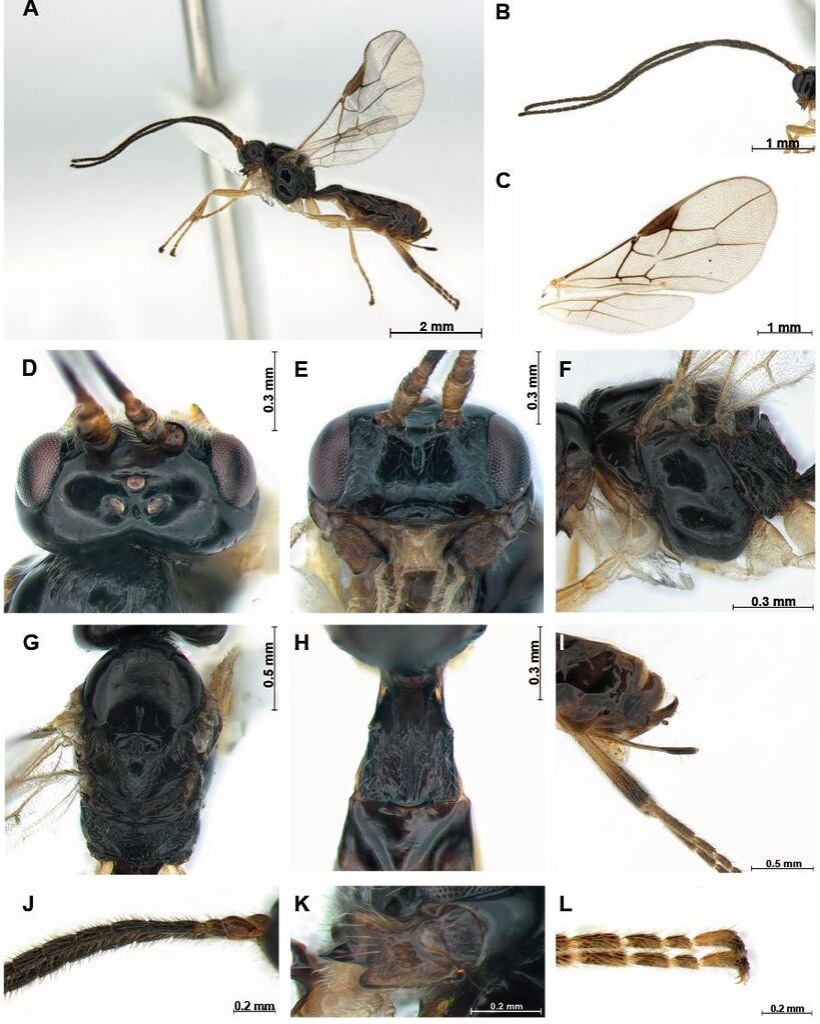
*Mesocrina indagatrix* Foerster, 1863. Female. **A** habitus, lateral aspect; **B** antennae; **C** wings; **D** head, dorsal aspect; **E** head, frontal aspect; **F** mesosoma, lateral aspect; **G** mesosoma, dorsal aspect; **H** first metasomal tergite, dorsal aspect; **I** ovipositor sheath, lateral aspect; **J** proximal part of antenna; **K** mandible, lateral aspect; **L** claw, lateral aspect.

**Female** (Fig. 2): length of body in lateral view 4.6 mm, length of antenna 5.0 mm and length of fore-wing 4.4 mm.

**Colour.** Body (Fig. 2) entirely black; antenna dark brown; mandible brown.

**Head.** Antenna (Fig. 2B) 1.1 times longer than body in female, 35-segmented. Fourth segment 0.8 times longer than third segment, 1.1 times longer than fifth segment. Width of face (Fig. 2E) 1.7 times its height from ventral rim of antennal sockets to upper margin of clypeus; face concave and one carina distinct medially; face with short setae; between eye and antennal socket concave and rugose. Eye in dorsal view 1.4 times longer than temple. Ocello-ocular line (OOL) 3.7 times longer than diameter of anterior ocellus; OOL: antero-posterior ocellar line (AOL) : postero-ocellar line (POL) = 26 : 7: 9. Stemmaticum concave and carina distinct. Mandible with three teeth and rugose (Fig. 2L); second tooth sharp and narrow, entirely reddish-brown, separated from first tooth; first tooth lobe-shaped. Clypeus 3.3 times longer than wide.

**Mesosoma.** Mesosoma 2.0 times longer than wide in dorsal view; 1.2 times longer than wide in lateral view. Mesosoma (Fig. 1G) with line-shaped medio-posterior depression; notauli weakly reduced posteriorly; anterior part of mesosoma rugose and setose; scutellar sulcus with one carina. Pronotum glabrous and entirely smooth; anterior part of mesoscutum with setae. Scutellum with dense and posteriorly directed setae. Maximum length of propodeum (Fig. 2G) 0.5 times its width; apical part of propodeum entirely rugose, medio-longitudinal carina present; propodeum without areola; precoⅹal sulcus (Fig. 2F) weakly developed; mesopleuron 1.6 times longer than wide. Fore-wing (Fig. 2C) 2.1 times longer than wide; pterostigma robust, 3.2 times longer than wide; vein r of fore-wing 0.4 times as long as wide; vein 2-SR: vein r: vein 3-SR = 5: 1: 7; vein 2–SR+M weakly sclerotised; vein SR1 1.6 times longer than vein 3-SR.

**Leg.** Hind coⅹa as long as hind trochanter; hind femur width 3.8 times median length in lateral view; 0.6 times as long as hind tibia, as long as tarsus. Arolium not longer than claw (1L).

**Metasoma.** First tergite entirely rugose, widened posteriorly, 2.1 times longer than its apical width. Setose part of ovipositor sheath (Fig. 2I) 0.4 times as long as mesosoma, 0.4 times as long as hind tibia and with short setae.

#### Diagnosis

Differs from other species in the group of *Mesocrina* species by setose part of ovipositor sheath about as long as first tergite, 0.4 times as long as hind tibia (0.7–0.9 times in other species); anterior half of mesoscutum with few setae, without punctures.

#### Distribution

South Korea, Austria, Bulgaria, China, Germany, Hungary, Netherlands, Spain, Sweden, Switzerland, United Kingdom (Yu et al. 2016).

### Alloea

Haliday, 1833

31A8D7CB-CCB5-598A-BF17-AFED079680E7

Alysia contracta (Haliday, 1833).

#### Diagnosis

Third tooth of mandible small, acute and shorter than elongate second tooth; in fore-wing, vein 1-SR of fore-wing distinct and in lateral view metanotum with truncate protuberance medio-dorsally; malar suture often rather long and deep.

#### Distribution

Palaearctic.

#### Biology (Host)

*Lonchoptera furcata* (Fallén, 1823) (Hedwig 1958), *Lonchoptera lutea* Panzer, 1809 (Smits van Burgst 1919).

### Alloea veles

Belokobylskij, 1997

5303CBFA-9EA8-525E-97EB-00F8CE8D59D0

PV546356

#### Materials

- **Type status:** Holotype. **Occurrence:** individualCount: 1; sex: female; lifeStage: adult; preparations: dry-specimen; occurrenceID: CC5581B4-DAE4-557C-90D7-5FF77F75CF32; **Taxon:** scientificName: *Alloea veles* Belokobylskij,1997; kingdom: Animalia; phylum: Arthropoda; class: Insecta; order: Hymenoptera; family: Braconidae; genus: Alloea ; specificEpithet: *veles*; scientificNameAuthorship: Belokobylskij, 1997; **Location:** higherGeography: East Asia; country: South Korea; countryCode: KR; stateProvince: Jeollabukdo; municipality: Namwonsi; locality: Mt. Segeolsan; decimalLatitude: 35.38533888888889; decimalLongitude: 128.5654; **Event:** year: 2022; month: 6; day: 20; **Record Level:** institutionCode: NIBR

#### Description

**Figure 3.**
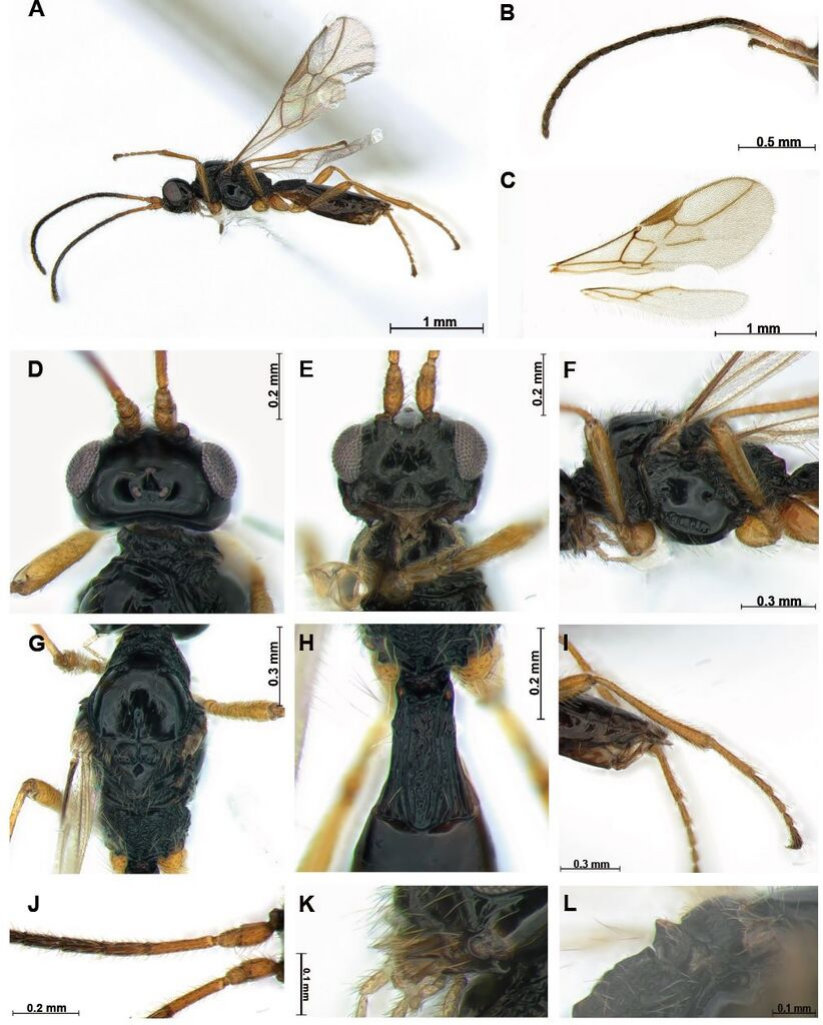
*Alloea veles* Belokobylskij, 1997. Female. **A** habitus, lateral aspect; **B** antenna; **C** wings; **D** head, dorsal aspect; **E** head, frontal aspect; **F** mesosoma, lateral aspect; **G** mesosoma, dorsal aspect; **H** first metasomal tergite, dorsal aspect; **I** ovipositor sheath, lateral aspect; **J** proximal part of antenna; **K** mandible, lateral aspect; **L** acute part of metanotum.

**Female** (Fig. 3): length of body in lateral view 2.5 mm, length of antenna 2.2 mm and length of fore-wing 2.2 mm.

**Colour.** Body (Fig. 3) entirely black; antenna dark brown, anterior part of segments yellowish-brown; mandible brown.

**Head.** Antenna (Fig. 3) 0.8 times as long as body in female, 23-segmented. Fourth segment 0.6 times longer than third segment, as long as fifth segment. Width of face (Fig. 3E) 1.5 times its height from ventral rim of antennal sockets to upper margin of clypeus; face concave medially; face with short setae; eye in dorsal view 1.6 times longer than temple. Ocello-ocular line (OOL) 4.6 times longer than diameter of anterior ocellus; OOL: antero-posterior ocellar line (AOL) : postero-ocellar line (POL) = 20 : 7: 7. Stemmaticum concave and carina distinct. Mandible with three teeth and rugose (Fig. 3L); second tooth sharp and narrow, entirely reddish-brown, separated from first tooth; first tooth short, sharp and narrow. Clypeus 1.8 times longer than wide.

**Mesosoma.** Mesosoma 2.0 times longer than wide in dorsal view, 1.5 times longer than wide in lateral view. Mesosoma (Fig. 3G) with line-shaped medio-posterior depression; notauli weakly reduced posteriorly; pronotum entirely rugose in dorsal view. Edge of the scutellum with dense posteriorly directed setae; precoⅹal sulcus (Fig. 3F) widely developed, not reaching to posterior part of mesopleuron; mesopleuron entirely glabrous, except few setae present ventrally; in lateral view, metanotum acute medio-dorsally (Fig. 3L). Maximum length of propodeum (Fig. 3G) 0.5 times its width; propodeum entirely rugose, medio–longitudinal carina absent; Propodeum without areola. Fore-wing (Fig. 3C) 2.7 times longer than wide; pterostigma robust, 3.7 times longer than wide; vein r of fore-wing 3.8 times longer than wide; 2-SR+M absent; vein 2-SR: vein r: vein 3-SR = 3: 1: 2.

**Leg.** Hind coⅹa as long as hind trochanter; hind femur width 5.6 times median length in lateral view; 0.7 times as long as hind tibia and as long as tarsus. Arolium not longer than claw.

**Metasoma.** First tergite entirely rugose, widened posteriorly, 1.4 times longer than its apical width; Setose part of ovipositor sheath short (Fig. 3I), 0.2 times as long as mesosoma, 0.2 times as long as hind tibia with short setae.

#### Diagnosis

Differs from other species in *Alloea kostaroma* by having robust vein r (slender in *A. kostroma*) and having robust pterostigma (slender in *A. kostroma*).

#### Distribution

South Korea, Russia (Belokoblylskij and Tobias 1997).

## Identification Keys

### Key to species of *Mesocrina* Foerster recorded from Korea

**Table:** 

1	Precoxal sulcus distinctly crenulate; posterior surface of propodeum sloping vertically; first tergite as long as its apical width; in fore-wing, vein 3-SR 3.5 times longer than r	***Mesocrina dalhousiensis* Sharma, 1978**
–	Precoxal sulcus weakly present; propodeum gradually sloping posteriorly; first tergite 1.4 times as long as its apical width; in fore-wing, vein r short, 3-SR 7.0 times longer than r	***Mesocrina indagatrix* Foerster, 1863**

### Key to species of *Alloea* Haliday from Korea

**Table:** 

1	Antenna 0.8 times as long as body; precoxal sulcus not reaching to posterior part of mesopleuron; vein r robust, 3.8 times longer than wide; pterostigma robust, 3.7 times longer than wide	***Alloea veles* Belokobylskij,1997**
–	Antenna 1.3 times longer than body; precoxal sulcus reaching to posterior part of mesopleuron; vein r slender, 5.8 times longer than wide; pterostigma slender, 7.1 times longer than wide	***Alloea kostroma* Belokobylskij,1998**

## Analysis

**DNA barcode (*COI*) analysis**

A total of 606 bp of the *COI* fragments were sequenced from *M. indagatrix* and *A. veles*, respectively, which were deposited in GenBank (accession numbers PV546355–PV546356) (Table 1). *M. chandleri* was used for distance comparison with *M. indagatrix*. In the case of *A. velves*, the genus *Alysia*, presumed to be a closely-related taxon, was included to assess genetic distance. Pairwise distances were estimated by using the P-distance model with the option for pairwise deletion. Interspecific distance ranged from 0.089 to 0.124 (average 0.131) (Table 2).

## Supplementary Material

XML Treatment for Mesocrina

XML Treatment for Mesocrina indagatrix

XML Treatment for Alloea

XML Treatment for Alloea veles

## Figures and Tables

**Table 1.** Species list for DNA barcode (*COI*) analysis for the present study.

No	Species	NCBI accession number	BOLD accession number	Reference
1	*M. indagatrix* Foerster, 1863	PV546355	-	this study
2	*A. veles* Belokobylskij, 1997	PV546356	-	this study
3	*M. chandleri* Godfray & van Achterberg, 2024	-	BRAAL477-19	Godfray and van Achterberg (2024a)
4	*Alysia erecta* Sohn & van Achterberg, 2023	OP391515	-	Sohn et al. (2023)

**Table 2.** Calculated genetics distance, based on COI sequences between species used in the analysis.

	*M. indagatrix*	*M. chandleri*	*A. veles*	*Alysia erecta*
*M. indagatrix*				
*M. chandleri*	0.089			
*A. veles*	0.112	0.114		
*Alysia erecta*	0.084	0.094	0.124	
